# Cases from the Note Book Robert Robson, M.D., New Harmony Ind.

**Published:** 1871-01

**Authors:** 


### MALPOSITION OF THE HEAD AS A CAUSE OF DIFFICULT LABOR.

Cases from the Note-Book of ROBERT ROBSON, M.D., New Harmony, Ind.

### CASE I.

On the 6th of January, 1868, I received an urgent message from Dr. V., in a case of obstructed labor, which he had been called to see in Canton Township, and, in which, from the extreme violence of the uterine action, he feared rupture of the uterus, if the head was not soon extricated. It was the woman's third labor, her previous ones having been natural, and in every sense favorable. In this instance, symptoms of labor had come on the evening before: the pains had been feeble during the night, but towards day became very active, and at 7 o'clock the head was pressing upon the perineum to such a degree that the expulsion of the child was momentarily expected, but there it remained without any advancement whatever when I arrived about 11 o'clock, although the uterus had been, during the whole of the intervening four hours, acting incessantly, and so powerfully, that its rupture was with some reason apprehended. On examination, I found the perineum and soft parts protruded by the head, they were unusually relaxed and yielding. I could pass my finger quite easily between it and the symplysis pubis, but at the sides there was no room—it seemed thoroughly wedged—the head lay transversly across the outlet of the pelvis, with the occiput resting against the tuberosity of the right ischium, and the forehead against the left; the head in such situation presents to the outlet the greatest possible dimensions which it is capable of assuming; its longest diameter resting its extremities on the opposite tuberosities of the ischia, while, at the same time, the parietal bones rest on the lower part of the sacrum and coccyx. Under such circumstances, the action of the uterus, however vigorous, seems totally incapable of effecting the delivery; the head could easily be raised into the cavity of the pelvis, but the next pain would force it back to its resting place. Under these circumstances, I proposed manual rectification of the displacement evidently existing, as I had done on similar occasions before, and having applied my two fingers along the sides of the forehead and parietals, I raised and pushed it backwards toward the right sacro-iliac symphysis, during the interval between the contractions of the uterus; I there retained it, and on the accession of the next two or three pains, I repeated the pressure backward, when soon afterwards the delivery was completed by the birth of a large, healthy-looking child.

It may be taken for granted, I presume, that every physician that has been engaged in obstetrical practice for any length of time, must have met, from time to time, with cases in which, while everything seemed favorable, and the labor apparently progressing to a desirable termination, when the head has become suddenly stationary in the cavity of the pelvis, and there remained for hours, or, perhaps, until a necessity has arisen for adopting instant means of delivery. The immediate cause of this species of arrest of labor, I am somewhat fearful is not as generally understood as it should be, though it requires only an intimate acquaintance with the mechanism of labor, and the exact relation which the different parts of the child's head bear to those of the pelvis during the progress of labor, and their application to practice; with such knowledge, we should, undoubtedly, often remove difficulties by means easy, safe, and effectual, instead of resorting to the use of instruments, the application of which, however skillfully used, expose more or less both mother and child to danger.

Some very excellent physicians, are unquestionably deficient in the practical knowledge of difficult parturition, and it undoubtedly arises, certainly not from any want of capacity, but from an aversion to this special branch of medical practice.

---

### RECTO-VAGINAL FISTULA.

---

CASE II.

Mrs. B., living in the town of New Harmony, having discharged her physician, sent for me in October, 1866.

On my arrival, I found the patient in the utmost distress imaginable, both mentally and bodily; the contents of the bowels passing per vagina, and attended with the most disgusting odor. After investigating the history of the case, I proceeded to place the patient in a proper position for examination; the parts being previously thoroughly cleansed. I then proceeded to introduce a Record-speculum, after removing one blade and the obturator, a light was brought to bear on the crown portion of the instrument, when the fistula, or communication between the rectum and the vagina became distinctly visible. The bowels having been freely opened before my arrival, I proceeded carefully to apply the argent. nitras to the edges of the perforated portion of the vaginal septum, after which, being aware of the very striking pathological distinction between mucous and serous surfaces, and the great difficulty of healing them when abraded or inflamed, I placed a wide slip of lint smeared with balsam copaib. carefully over the fistula to guard against the presence of mucus. The difficulty of healing, in such cases, does not arise so much from peculiarity of structure, as from the circumstance of the presence of mucus, which will not mix or amalgamate with the fibrin of the blood, and, therefore, prevents the formation of that bond of union which is essensial to adhesion.

It is well-known to some, that the mucous discharge which is constantly running over the breach of continuity, is the cause why these injuries are so difficult to heal.

From the happy termination of the above case, the patient recovering in ten or twelve days, her evacuations passing the regular way, I have but little doubt the treatment will be found successful in lacerations or abrasions of mucous surfaces generally.

Throughout the whole treatment, the patient was sustained with small potions of liquid diet, and the bowels maintained in comparative rest.

---

### MONSTROSITY.

### CASE III.

On the 8th day of October, 1868, I was called to attend on Mrs. A., of Bishell township, Posey Co., the wife of a respectable farmer, and the mother of two lovely children. She had reached the full term of pregnancy. She was in labor on my arrival, the head presenting, her pains natural and effective, and, after a few hours, she was delivered of a living male child, of ordinary size, which, after a few respirations, expired. On examination, the posterior and superior portion of the brain was found soft, and covered only by the scalp, the parietal bones only partially ossified, and the entire os occipital wanting. The inferior extremities were also defective; the right was perfect, while the left terminated at the inferior portion of the femur, which was enlarged, forming a somewhat analogous appearance to the opposite knee-joint, and from which projected a foot, perfect in structure and of the same size as its fellow.

Such, and similar cases to the above must necessarily fall into the hands of every practising physician of long standing, and are, unfortunately, passed over by many without apparent notice or remark, either unwilling or too busy to spend time in the acquirement of pathological anatomy, which alone can enable them to detect the various morbid derangements of any, or of all the parts of the system; to trace their origin and explain the processes by which such monstrosities are produced. It is well known that many attribute much influence over the growth of the fœtus in utero to the imagination of the mother, something for which the mother has longed without being able to get it, or to some fright or object of terror, which is supposed to disturb the natural process of gestation, and mark the unborn child, etc. Such impressions are prevalent among the more ignorant portion of the people, and, unfortunately, some physicians of the present day entertain such antiquated notions. The perversions or lesions of nutrition, their metamorphic changes, etc., are, by such persons seldom thought of in reference to morbid alterations of structure, or monstrosities. Irregularities, or deficiencies of nutrition are often consequent on the distribution of the arteries and nerves in the embryo child; for wherever healthy blood is sent, accompanied with the necessary nervous stimulus, nutrition must be accomplished and the fœtus perfected, whereas any excess or deficiency of either must result in deformities.

Some one of our distinguished authorities, (I do not recollect who), maintains that monstrosities are connected with, or consequent upon, deficient parts of the nervous system; that with the absence of any nerve there is connected the absence of the organ to which the nerve belongs; and with the imperfect formation of any part of the nervous system, there is deposited the imperfect development of the organ which it supplies. If I am not incorrect, that same authority maintains that the nervous system is the first existing apparatus in the formation of the fœtus, and regulates the formation of the embryo, and determines the form. If these views are correct, we can have no further difficulties in accounting for monstrosities.

### PLACENTAL DISEASES.

CASE IV.

I was called to attend on Mrs. D., of Lynn township, Posey county, in her labor some time since, when, on my arrival, I ascertained that she had been in labor nearly six hours, and was much exhausted. Her pulse was rapid, rather small and weak. She complained of much pain and irritation of the whole uterine region, but, notwithstanding her labor pains were very inefficient, not more than every third pain apparently doing any good, she was delivered, in four hours after my arrival, of twins, one of them being a living, healthy-looking boy-child, and the other dead and in a state of putrefaction. The placenta belonging to the living child was of a healthy and firm consistence, while that of the dead child was apparently a cartilaginous mass, some portions of which were thoroughly ossified. During an obstetrical practice of about forty years, I have seen similar conditions of the placenta. In cases of this kind, the cause of death in still-born children is seldom inquired into, and there is, perhaps, no branch of pathology involved in greater obscurity than that connected with the fœtus and its appendages in utero. Death is generally attributed to some disease affecting the child itself, neither understood or sought after, or else to a separation of a part or the whole of the placenta from the surface of the uterus. It is seldom that the condition of the placenta itself is examined, and as seldom that the child undergoes any inspection. That the placenta is liable to disease like every vital part, is abundantly proved by the fact that a part is not unfrequently found converted into cartilage. It may fairly be inferred that the placenta is subject to other diseases, and that these diseases must frequently be the cause of the death of the fœtus, and of much suffering on the part of the mother. It is, perhaps, true that very few cases are attended with any difficulty, and that a certain scientific delivery of the child will generally secure a safe and prompt expulsion of the secundines, but this surely is no reason why the diseases of the placenta should not be more minutely and scientifically examined, since it is only by viewing these subjects as presented by different practitioners, and under many phases, that we can hope to acquire knowledge of the confused and irregular operations of nature.

What morbid process or modification of nutrition the placenta undergoes before it is converted into bone, I am not prepared to say, though no doubt it succeeds much irritation or increased vascular action of the part implicated; yet in the above case no morbid action, or cartilaginous or osseous transformation was felt or suspected up to the time of the expulsion of the placenta.

---

### ON SULPHATE OF MORPHINE AND ERGOT AS PARTURIENTS.

---

CASE V.

SULPHATE OF MORPHIA.—I have had frequent occasion to administer this article in labor, in cases of nervous irritability, with a view to its anodyne effect, when it has resulted in producing effects similar to the Secale Cornutum. Time after time I have realized this result. It is my impression that its *modus operandi* is to cause an increased irritability of the excito-motory nerves. I have used it in the absence of the pulv. ergotæ with equal success.

Dr. John Beal, of this place, for several years a practitioner, states that he has often witnessed the parturient effect of sulp. morphine, when its anodyne property was both desired and anticipated. We think that the parturient effect of this article must undoubtedly have been noticed by others, and we should like to hear from them on the subject. Such a property in the sulph. morphia would undoubtedly account for the many failures in the prevention of abortion where opiates have been given.

ERGOT.—Much has been written in reference to tbe deliterious consequences resulting from this article, when employed to facilitate labor.

I have had occasion frequently, during a large obstetrical practice of forty years in this locality, to use the pulv. ergotæ with a view to the expulsion of the fœtus, or to expel the hydatiform masses developed in the cavity of the womb, and, strange as it may appear, have never realized any bad results from it. I have never administered it before the os uteri was considerably dilated and the external parts relaxed, but most assuredly, wherever it acted at all, the termination of the labor was all right. I have been the more astonished at this, as I am aware of the abundance of testimony in favor of opposite results. I have often administered the article and repeated the dose without any effect on the uterus having followed, and therefore concluded that either the medicine was inferior in quality or the constitution of my patient was not susceptible to its action, yet, at other times, I have given the same ergot, when soon after, the pains would be severe and continuous until labor was completed, making it evident that some constitutions were not susceptible to its impression.

Some physicians doubt the efficiency of ergot in producing contractions of the uterus.

That the failure of this article in numerous cases, is attributable, either to the inferiority of the article used, or the want of susceptibility of the patient to its impressions, is very evident to me. That it acts promptly in many cases is also evident from the very different character of the labor-pains from those of ordinary labor—the one severe and continuous—the other more regular and intermittent. The failure of ergot, is, undoubtedly, often attributable to its inferior quality. While in England I witnessed its growth, and know that it is effected by a variety of causes influencing the season; a moist or wet spring and summer facilitates its growth, while a dry autumn secures its activity. If collected before the harvest, it was considered very energetic, and possessing the property of promoting uterine contractions, as well as preventing uterine hemorrhage; whilst that which was picked after harvest had little or no activity. This may explain, in some degree, the great diversity of opinion in reference to the reliability of this article.

